# RNA editing enzymes: structure, biological functions and applications

**DOI:** 10.1186/s13578-024-01216-6

**Published:** 2024-03-16

**Authors:** Dejiu Zhang, Lei Zhu, Yanyan Gao, Yin Wang, Peifeng Li

**Affiliations:** 1 Institute for Translational Medicine, College of Medicine, The Affiliated Hospital of Qingdao University, Qingdao University, Qingdao, China; 2 College of Basic Medical, Qingdao Binhai University, Qingdao, China

**Keywords:** RNA editing, ADARs, APOBECs, Deaminase, Immunity

## Abstract

With the advancement of sequencing technologies and bioinformatics, over than 170 different RNA modifications have been identified. However, only a few of these modifications can lead to base pair changes, which are called RNA editing. RNA editing is a ubiquitous modification in mammalian transcriptomes and is an important co/posttranscriptional modification that plays a crucial role in various cellular processes. There are two main types of RNA editing events: adenosine to inosine (A-to-I) editing, catalyzed by ADARs on double-stranded RNA or ADATs on tRNA, and cytosine to uridine (C-to-U) editing catalyzed by APOBECs. This article provides an overview of the structure, function, and applications of RNA editing enzymes. We discuss the structural characteristics of three RNA editing enzyme families and their catalytic mechanisms in RNA editing. We also explain the biological role of RNA editing, particularly in innate immunity, cancer biogenesis, and antiviral activity. Additionally, this article describes RNA editing tools for manipulating RNA to correct disease-causing mutations, as well as the potential applications of RNA editing enzymes in the field of biotechnology and therapy.

## Backgrounds

RNA can undergo a variety of modifications, but only a small proportion of modifications lead to changes in RNA base pairing, that is, RNA editing [1–3]. The discovery of RNA editing began with studies of the biochemical reactions of adenosine deamination and recoding events in the mammalian GRIA2 transcript encoding GluA2 (glutamate ionotropic receptor AMPA-type subunit 2) [4, 5]. Melcher and colleagues compared mouse brain genomic DNA to cDNA and found that GRIA2 is a site in the genome that changes to G in mRNA, resulting in a codon change from glutamine to arginine at the corresponding position. Later studies proved that this phenomenon was not caused by genome mutations but by RNA editing [6]. The enzyme that performs this editing is ADAR2, which catalyzes the hydrolytic deamination of A (adenosine) at C6 to I (inosine), which is recognized as G when the codon is decoded because it pairs similar with G. Compared with the biological functions of RNA editing discovered later, the function of RNA editing discovered here is groundbreaking; therefore, the study of RNA editing has indeed become an important part of the neurobiology of neurological function and psychiatric disorders [7]. Interestingly, some RNA editing sites are highly conserved across species, for example, RNA editing of potassium channels is highly conserved from insects to squid [8]. Since then, interests in RNA editing have grown. Up to now, there are ADAR proteins have been found in mammalian genomes. These include ADAR1, which has two subtypes (ADAR1p110 and ADAR1p150), ADAR2 (*ADARB1*), and ADAR3 (*ADARB2*) [4, 9]. Among these proteins, ADAR1 and ADAR2 are believed to be active, while ADAR3 functions as an inhibitor of A-to-I editing. There are two other classes of RNA editing enzymes. APOBECs (apolipoprotein B mRNA editing catalytic polypeptide-like family) catalyze cytidine to uridine (C-to-U), and ADATs (adenosine deaminases acting on transfer RNAs), which are primarily responsible for A-to-I editing events on tRNAs [9, 10].

The process of RNA editing can occur in various types of RNA molecules, including pre-mRNA, mature mRNA, miRNA, lncRNA, tRNA, and even viral RNA [11–13]. An important impact of RNA editing is its ability to challenge the central dogma of biology. When RNA editing takes place in the coding sequence (CDS) region of an mRNA, where an I is recognized as a G or a C as a U after deamination, it can lead to amino acid substitutions (called ‘recoding’) and enhance the diversity of the proteome. However, it is important to note that RNA editing is primarily carried out in noncoding regions of mRNAs or noncoding RNAs. The primary role of ADAR1 editing is to modify the structure and immunogenicity of cellular double-stranded RNA (dsRNA). Recent studies have indicated that the recoding occurs in various genes with similar levels among most tissues, without being particularly abundant in the brain [14–16]. The levels of RNA editing are intricate in different cells and cannot be easily categorized [17]. For instance, certain genes (CCNI, COPA, and AZIN1) exhibit more alterations in endothelial cells compared to excitatory neurons, while TMEM63B shows less alteration in endothelial cells [17, 18]. It has demonstrated that ADARs play crucial roles in brain development, viral defense mechanisms [19–22], and various human diseases, including cancer [23–30], autoimmune diseases [23, 31, 32], autoinflammatory diseases [24, 33], atherosclerosis [34, 35], and heart failure [36]. Recent studies have also revealed the significance of ADAR2 in tissue inflammation through its control of the IL-6 signaling pathway [37]. Therefore, further exploration of RNA editing not only deepens our understanding of its biological role, but also paves the way for the development of tools that exploit RNA editing mechanisms to treat diseases. This review article aims to investigate the mechanism of RNA editing, its relationship to disease, and the potential application of tools based on RNA editing enzymes in disease treatment.

## A-to-I RNA editing

The ADAR protein family, functions as RNA editing enzymes, was initially identified as the enzyme responsible for “denaturing” dsRNA in *Xenopus laevis* embryos, unintentionally interfering with RNAi experiments [38]. ADARs are believed to have evolved from ATADs, which are present in yeast and mammals and have a bacterial counterpart called TadA [11, 39, 40]. Adat catalyzes A-to-I RNA editing reaction specifically in tRNA.

### Structural characteristics of ADARs

**Fig. 1:**
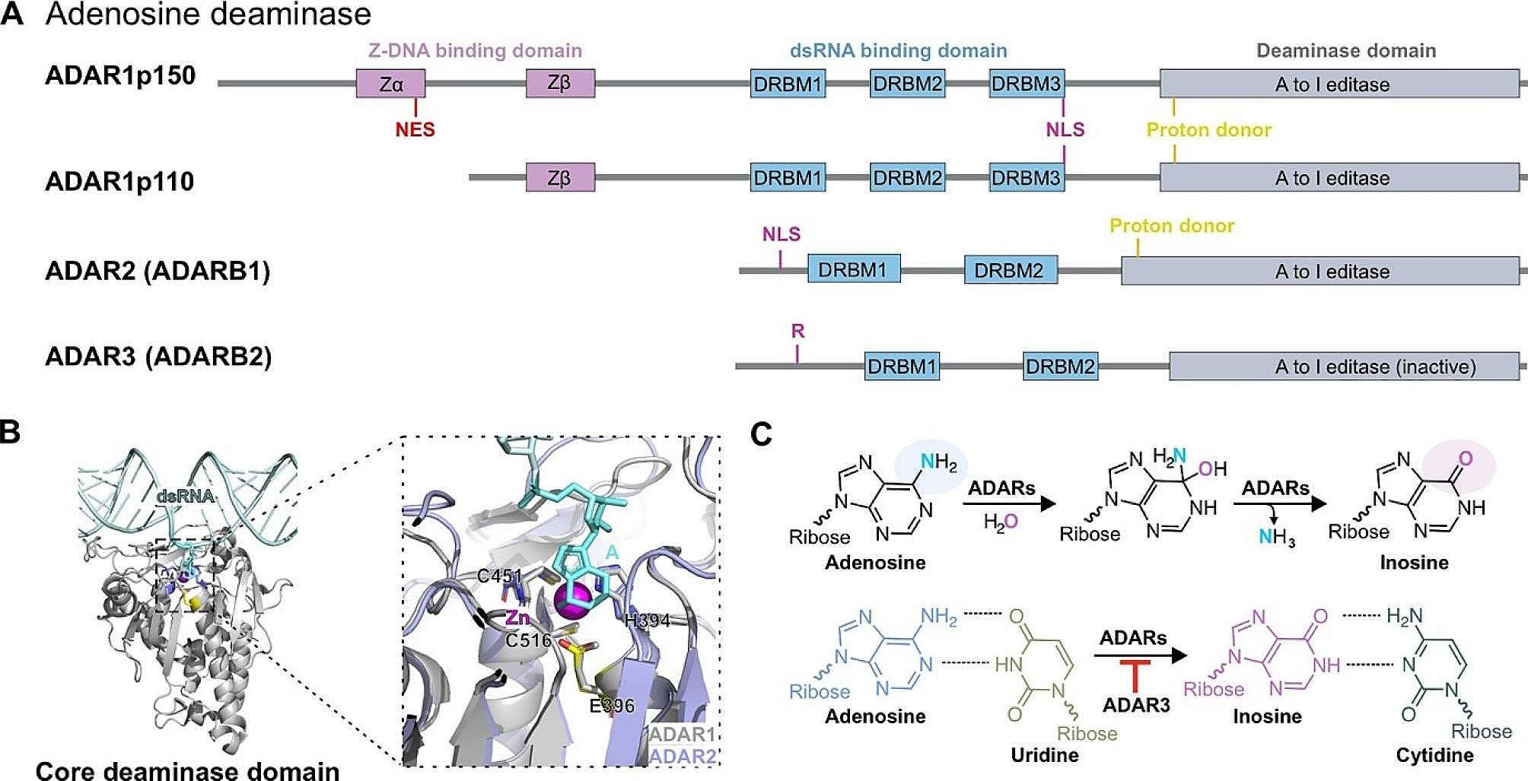
Structural characterization and catalytic mechanism of RNA adenosine deaminase. **A** Organization of domains in ADAR family proteins. **B** The deaminase domains of ADAR1 and ADAR2 are structurally similar. (PDB: 8E4X). **C** Schematic representation of the mechanism of ADARs catalyzed deaminase. Removal of the C6 amino group allows inosine to base pair with cytosine. Zα, Z-RNA binding domain; NES, nuclear export signal; NLS, nuclear localization signal

ADAR family proteins are highly conserved and have a similar domain arrangement (Fig. 1A and B). The C-terminal region contains the catalytic deaminase domain. Among these proteins, only ADAR1 and ADAR2 exhibit deaminase activity, while ADAR3 lacks a functional deaminase domain [41]. The upstream region of the catalytic domain contains two to three dsRNA-binding domains (dsRBDs), indicating that the binding of ADARs to RNA is not specific to a particular sequence, but rather to the structure dsRNA (Fig. 1B). The p110 isoform of ADAR1 is constitutively expressed in major human tissues whereas, the long isoform of ADAR p150 is expressed from an interferon (IFN)-inducible promoter. The extended N-terminal region found in the ADAR p150 isoform serves as the nuclear export signal (NES) and Z-DNA binding domain. Although both ADAR1 p110 and ADAR1 p150 can move between the nucleus and cytoplasm, p110 is predominantly located in the nucleus, while p150 is primarily present in the cytoplasm [9]. The functional differences between p110 and p150 in cells correspond to their respective localizations. ADAR2 possesses two dsRBDs and a deaminase domain and it is mainly involved in editing the coding regions of central nervous system transcripts. ADAR2 is a core protein that can shuttle between the nucleolus and nucleoplasm through expression and substrate binding [23, 42]. ADAR2 autoregulates its expression and activity by editing its own pre-mRNA to create 3’ splice sites, resulting in reduced expression of functional ADAR2. ADAR3, the third member of the ADAR protein family, is specifically expressed in certain regions of brain [43–45]. ADAR3 shares 72% sequence similarity with ADAR2 and possesses two dsRNA-binding motifs and a deaminase catalytic domain. Although ADAR3 does not exhibit RNA editing activity, it may function as a dsRNA-binding protein and regulate the binding of the other two ADAR proteins to dsRNA, thereby affecting RNA editing (Fig. 1C).

### Molecular functions of A-to-I editing

**Fig. 2:**
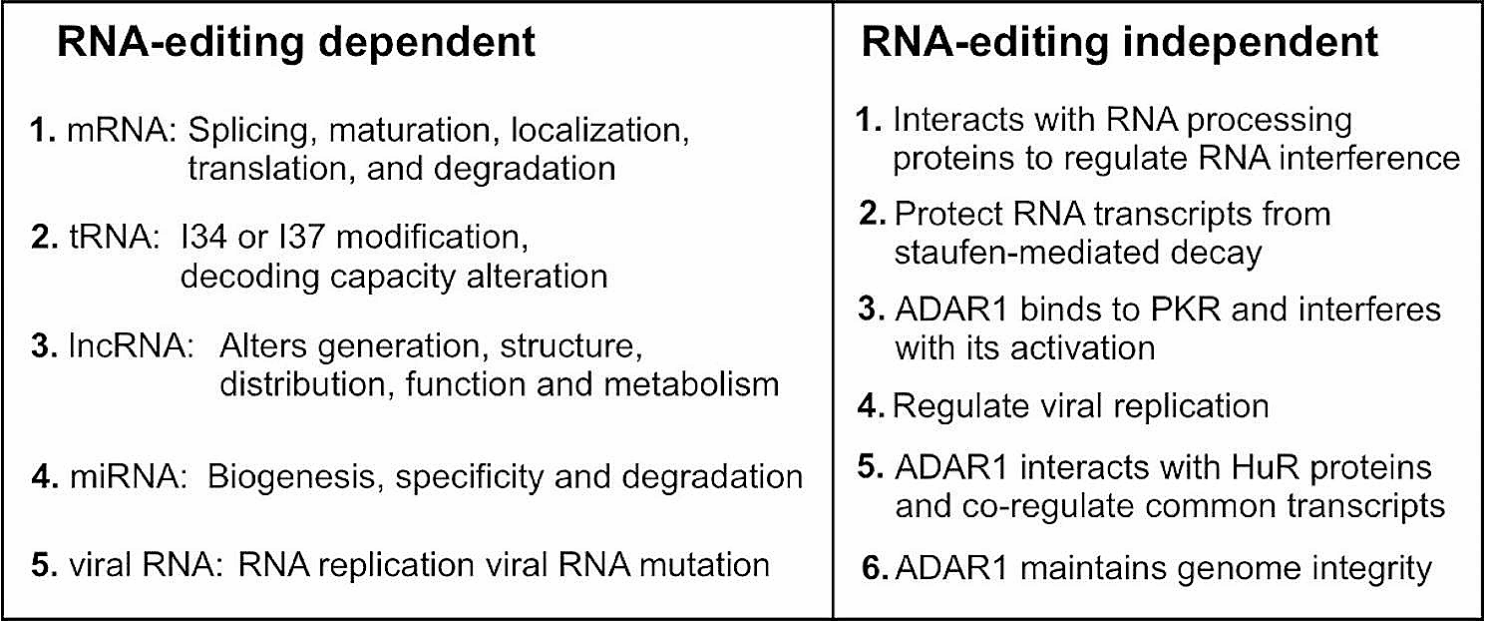
Functional consequences of RNA editing enzymes

ADARs deaminate adenosine in dsRNA or the A-form helix (DNA: RNA hybrid) [46–48]. The introduction of non-canonical base pairs into the dsRNA structure increases complexity, which actually affects the efficiency of adenosine editing (Fig. 2). The two bases next to the edited adenosine in the RNA sequence play an important role in the efficiency of RNA editing [49]. RNA editing is more likely to occur when the edited adenosine is preceded by a pyrimidine and followed by a G (e.g., 5’-UAG-3’) [17, 50]. In addition to adjacent nucleotides, mismatches at processing sites can also increase the processing efficiency. Classical A-U pairings can be edited, with A-C mismatches being the most efficient among adenosine mismatches, while A-A or A-G mismatches are the least efficient [2]. Therefore, the preferred neighboring nucleotides and the presence of an A-C mismatch at the editing site contribute to the efficiency of dsRNA deamination by ADARs.

RNA editing plays a crucial role in the diversification of protein isoforms and functions within the mRNA coding sequence. However, relatively few edits have been made to the protein-coding regions of mammalian transcripts, with only 40 conserved positions identified [17, 51]. In contrast, the majority of A-to-I RNA editing events occur in noncoding RNAs, introns, and 3’ UTRs in both humans and mice [52–54]. One intriguing aspect of ADAR1 biology is its association with Alu elements, which are derived from the duplication of short interspersed nuclear elements in humans [16, 55]. These Alu elements, which consist of two opposite-oriented repeat elements, form a double-stranded hairpin structure that is commonly targeted for RNA editing [34, 56, 57]. Although Alu elements are predominantly found in introns and untranslated regions of genes, they are occasionally found in translated regions as well. ADARs are capable of recognizing and binding this RNA hairpin, leading to the conversion of the specific A-to-I through hydrolytic deamination through hydrolytic deamination [44]. A-to-I cotranslational editing events can result in intron retention or exonization, as the removal of splice sites results in the expression of different protein isoforms. For instance, A-to-I editing on intronic Alu elements can convert the canonical 5’ splice donor site AU to IU and/or the canonical 3’ splice acceptor site AA to AG, potentially impacting the splicing of transcripts [58–60]. The editing process causes IU to form wobble base pairs in the paired regions of double-stranded RNA, disrupting dsRNA pairing and causes the dsRNA structure to expand [30, 61]. In human cells, endonuclease V utilizes Tudor-SN nuclease as a cofactor to degrade edited dsRNA [62].

Previous studies have demonstrated that the export of some overedited RNA to the cytoplasm is inefficient, resulting in inosine-containing RNA being retained in the nucleus. However, another study conducted in *C. elegans* and humans revealed that neither dsRNA formation nor A-to-I editing affects RNA location. These contradictory findings raise the question of what effect A-to-I editing has on RNA nuclear retention. A-to-I editing also plays a role in regulating the stability of RNA structures. Substituting A with I in an A-U base pair reduces stability, while introducing I in an A-C mismatch increases stability [63]. Wone et al. demonstrated that A-to-I editing of the 3’ UTR of stearoyl-CoA desaturase (SCD1) increased KHDRBS1 binding, thus enhancing the stability of SCD1 mRNA [64]. Apart from ADAR1, ADAR2 also binds to and stabilizes RNA in a manner that is independent of editing. RNA editing can also be observed in microRNAs (miRNAs) [65]. In the nucleus, primary miRNAs generate miRNAs during the formation of hairpin dsRNA structures that are targeted by ADARs, and A-to-I editing can impact various steps of miRNA biogenesis [66]. ADARs not only compete for binding to miRNA precursors but also interact with RNAi processing components, thereby regulating the efficiency of miRNA maturation. A-to-I editing can also affect RNAi by altering the miRNA-binding site within the mRNA and the specificity of the miRNA. The ability of lncRNA to form dsRNA structures makes it a potential ADAR substrate, and A-to-I editing can influence the stability and function of lncRNA by altering the structure of lncRNA and its interaction with miRNA [53]. This suggests a connection between RNA editing and gene silencing in terms of impeding gene regulation.

### C-to-U RNA editing

**Fig. 3:**
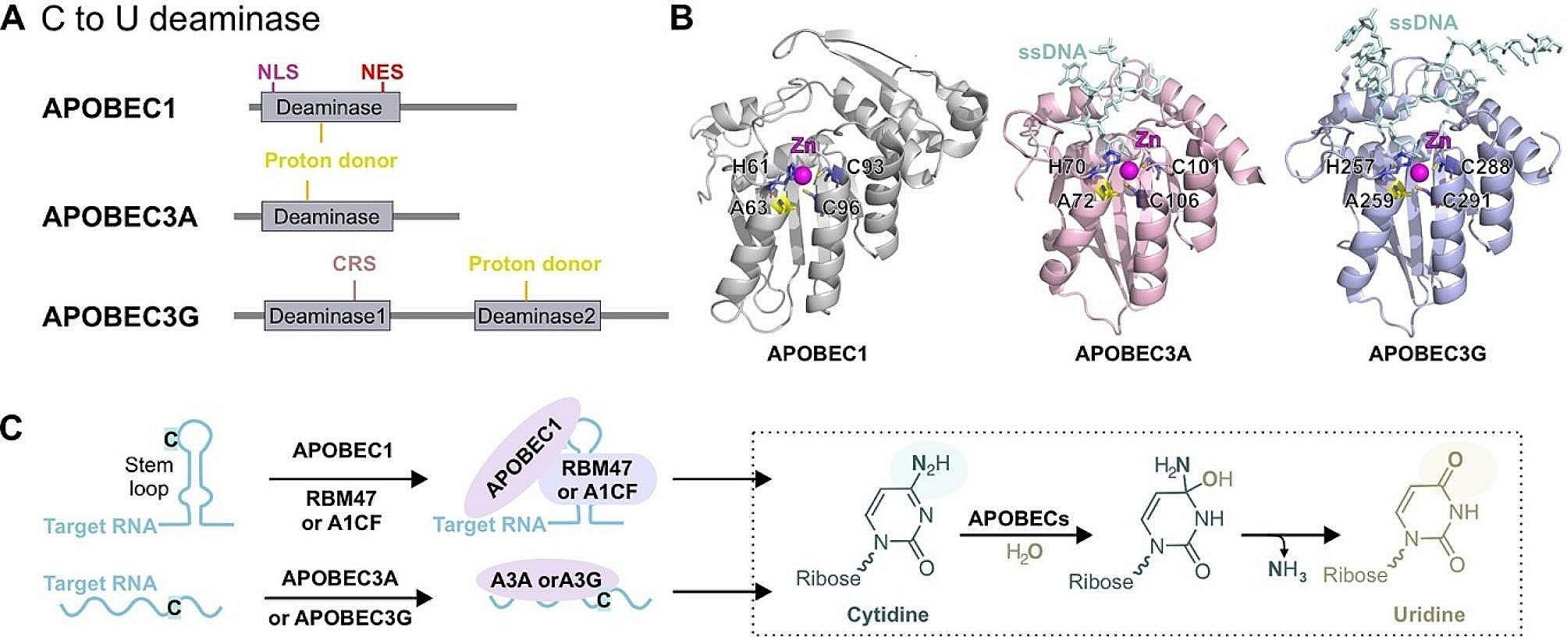
Structural characterization and catalytic mechanism of cytosine deaminase. **A** Organization of domains in cytosine deaminases. **B** The structure of core deaminase domains. (APOBEC1 PBD: 6 × 91, APOBEC3A PDB: 5KEG and APOBEC3G PDB: 6BUX) **C** The deaminase function of APOBEC1 depends on its binding to RBM47 or A1CF, while APOBEC3A and APOBEC3G can directly catalyze the C-to-U reaction with deaminase activities. RBM47, RBM4RNA binding motif 47; A1CF, Apobec1 complementation factor

Cytidine deaminase is an enzyme that converts C-to-U through a hydrolytic deamination reaction (Fig. 3). It is found in both prokaryotic and eukaryotic systems and has the ability to deaminate cytidine in both DNA and RNA. APOBECs, a class of enzymes that perform cytidine deamination, include APOBEC1, APOBEC2, APOBEC3A-H, APOBEC4, and activation-induced cytosine deaminase (AICDA) [67, 68]. In humans, APOBEC A3 enzyme family consists of seven enzymes: A3A, A3B, A3C, A3D, A3F, A3G, and A3H. These genes are highly conserved in vertebrates, with APOBEC1 and A3 found exclusively in mammals [69]. Although they all possess similar zinc-dependent deaminase domains, only APOBEC-1, APOBEC-3 A, APOBEC-3B, APOBEC-3G, and AICDA exhibit cytosine deaminase activity [1, 70]. These enzymes are known to be expressed in macrophages, monocytes, and NK cells under hypoxia and IFN stimulation and to play a role in the immune system. Interestingly, the cytosine deaminase action of APOBEC family proteins was initially observed on single-stranded DNA and genomic DNA, and we have a better understanding of APOBEC DNA editing compared to APOBEC RNA editing [67, 70]. This article mainly focuses on the RNA editing function of APOBEC family proteins. In addition to mammals, there is evidence of C-to-U RNA processing in plant mitochondria, suggesting a biologically important function [69]. APOBEC1 was first identified through the discovery of a C-to-U modification in apolipoprotein B (ApoB) mRNA, which results in the expression of two distinct forms of the protein (a truncated form and a full-length form) [71, 72]. The full-length ApoB protein is responsible for the cholesterol transport, while the truncated ApoB protein is responsible for the transport of triglyceride in the blood [42, 71, 73]. Similar to ADARs, APOBEC family proteins mainly target noncoding and intronic regions of transcripts containing Alu elements. APOBEC proteins have been shown to inhibit retroviruses, endogenous retroelements, and other viruses in a manner that is dependent or independent of their RNA editing function.

### A-to-I editing of tRNAs

**Fig. 4:**
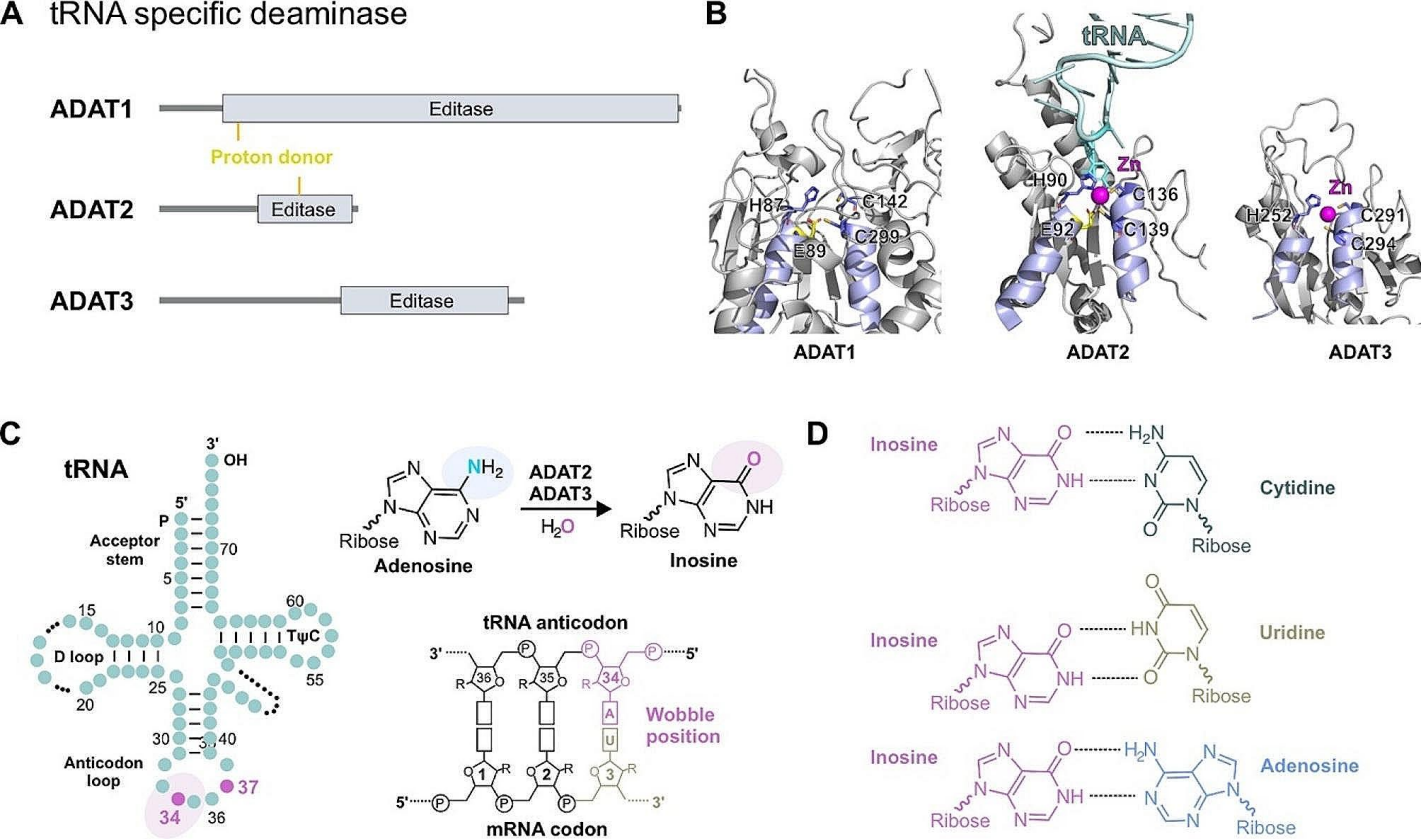
Structural features and catalytic mechanism of ADATs. **A** Organization of domains in ADAT family proteins. **B** The structures of core domain of human ADATs. The structure of ADAT1 is derived from Alpha fold, while the PDB number of ADAT2 and ADAT3 is 8AW3. **C** Deamination of adenosine at position 34 (wobble position) of tRNA, catalyzed by ADAT2 and ADAT3. ADAT3 has a core domain similar to ADAT2, but lacks a proton donor and thus has no catalytic activity. **D** Inosine 34 locates in the wobble position, which can pair with cytidine, uridine, or adenosine (base pairs ability: C > U > A)

In eukaryotes, ADATs catalyze the A-to-I modification by substituting adenosines with inosines at specific positions within the tRNA [8, 74, 75] (Fig. 4). In bacteria, inosine 34 is catalyzed by a homodimer of TadAs (bacterial tRNA deaminase). In eukaryotes, adenosine can be converted to inosine at positions 37 and 34 of tRNA [75]. ADAT1 catalyzes the adenosine modification at position 37 of tRNA^Ala^_AGC_ [11]. In eukaryotes, the heterodimers ADAT2 (active) and ADAT3 (inactive) may be involved in the deamination of adenosine 34 to inosine in many tRNAs [76]. These ADATs carry the cytidine deaminase (CDA) active motif (C/H)XEXnPCXXC (with X being any amino acid, and n being any number of residues) [76, 77]. In eukaryotes, I34 is present in eight tRNAs (tRNA^Thr^_AGT_, tRNA^Ala^_AGC_, tRNA^Pro^_AGG_, tRNA^Ser^_AGA_, tRNA^Leu^_AAG_, tRNA^Ile^_AAT_, tRNA^Val^_AAC_ and tRNA^Arg^_ACG_) [75, 78]. The position 34 of tRNA is particularly important as it is the first nucleotide of the anticodon loop, known as the wobble position, which pairs with the third position of the base triplet in the mRNA [79]. The A-to-I editing of adenosine 34 improves the decoding ability with a single modification, since the inosine base pairs not only with C but also with A or U (pairing C > U > A), thereby increasing the number of codons recognized by the tRNAs [80]. However, the mechanism by which loss of tRNA wobble A to I modification affects disease remains to be investigated. Furthermore, the impact of tRNA defects deamination on tRNA stability, abundance, maturation, aminoacylation, and protein translation also needs to be explored. It has been reported that inosine 34 in tRNA affects the regulation of gene expression during pluripotent stem cell differentiation by improving translation efficiency [79, 81].

## Biological functions of RNA editing

Knockout of ADAR genes in HEK293 cells induces interferon production, which is thought to be caused by endogenous non-editing dsRNA that is recognized by cellular machinery as viral genetic material [82]. Complete loss of either ADAR1 or ADAR2 can lead to spontaneous death [83]. ADAR1-null mice die on embryonic day 12.5, while ADAR2-null mice die either before or at weaning around P20 [84]. Even mice deficient in ADAR1-E816A/E816A editing die in utero [85]. ADAR2 is essential for editing the neuronal glutamate receptor Gria, and only the edited Gria2 pre-mRNA can be efficiently spliced, while the unedited pre-mRNA remains in the nucleus [4]. The insertion of newly encoded Gria2 rescue the mice suggests that Gria2 editing is a crucial survival function of ADAR2 [4, 86]. ADAR1 has also been found to mediate recoding events of potential physiological significance, but its primary role in mammals has not yet been confirmed [84]. Finally, ADAR3-KO mice were mostly normal but showed cognitive deficits in learning and memory, while global A-to-I editing remained largely unchanged [87].

### RNA editing in immunity

**Fig. 5:**
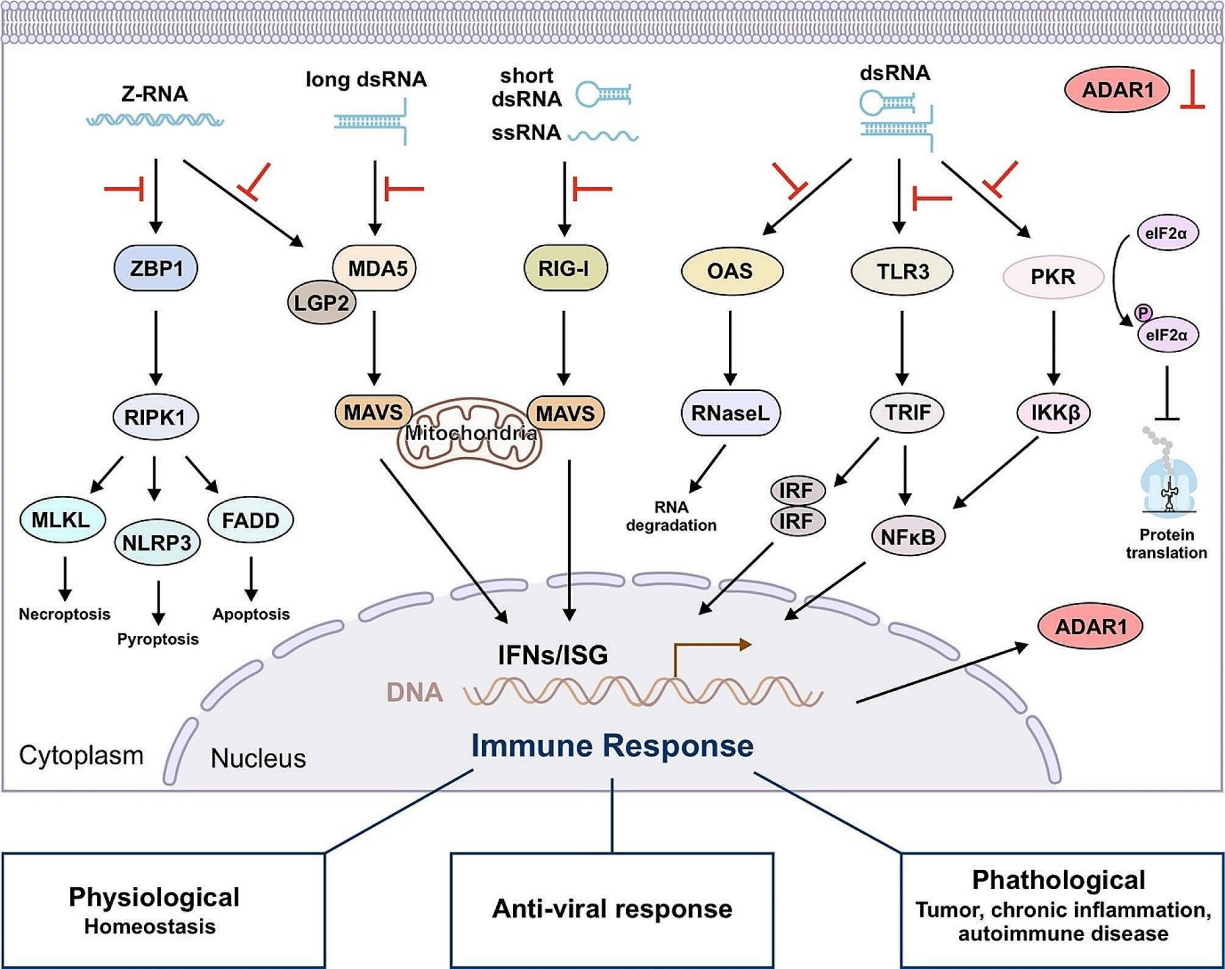
The RNA editing enzyme ADAR1 plays a crucial role in innate immunity. Double-stranded RNA (dsRNA), whether endogenous or exogenous, can bind to cytoplasmic RNA receptors and trigger an innate immune response. The ADAR1 catalyzed A-to-I RNA editing can suppress these immune responses by disrupting RNA structure

Furthermore, ADAR1 has been identified as a key player in the innate immune response [30, 88, 89]. Host RNAs undergo modification catalyzed by ADARs (A-to-I editing) to avoid potentially pathological IFN signaling and PRR (pattern recognition receptor) sensing by endogenous dsRNA [90, 91]. Mitochondrial antiviral-signaling protein (MAVS) plays a crucial role in signaling pathways that maintain immune homeostasis and antiviral responses [92]. Pattern recognition receptors such as RIG-I (retinoic acid-inducible gene I), MDA5 (melanoma differentiation-associated gene 5), and TLR (toll-like receptors) recognize dsRNA or ssRNA and trigger MAVS oligomerization. This in turn activates downstream factors and triggers interferon response [30, 93, 94]. These receptors recognize specific features of RNA structure. RIG-I recognizes the ends of dsRNA, while MDA5 recognizes the internal duplex of RNA [95, 96]. A-to-I editing converts A-U base pairs in the dsRNA region into I-U mismatches, thereby disrupting the RNA duplex structure. This suggests that ADAR1 and A-to-I editing, mediated by MDA5 polymerization to dsRNA and MAVS activation, play a crucial role in suppressing IFN production and preventing aberrant innate immune responses [24, 33, 84] (Fig. 5).

Interferon-induced p150 isoforms are thought to play a role in this signaling pathway, potentially through editing specific targets of p150 that can be acquired through their cytoplasmic localization and/or additional Z-DNA/RNA-binding domains [97–99] (Fig. 5). This is supported by the fact that the embryonic lethality of ADAR1 deletion can be overcome by a second knockout of MAVS or MDA5/Ifih1, indicating that ADAR1 plays a critical role in the activation of the dsRNA recognition pathway and suggesting a role for innate immunity. ADAR1 mutation leads to Aicardi-Goutières syndrome (AGS), a severe autoinflammatory disorder associated with abnormal IFN production [100–102]. In addition to inhibiting the MDA5/MAVS axis, ADAR1 also regulates PKR (protein kinase R) activity [96, 103–105]. PKR is another dsRNA sensor involved in antiviral response, triggering translational shutdown and apoptosis through phosphorylation of eIF2α (eukaryotic initiation factor 2α) [101, 106, 107]. Furthermore, ADAR1 can inhibit the activation of the oligoadenylate synthetase (OAS)/ribonuclease (RNase) L signaling pathway, which involves recognition of dsRNA by OAS and subsequent activation of RNase L [88, 108]. The only other mammalian molecule containing a Zα domain is ZBP1 (Z-DNA binding protein 1) [46, 109]. ADAR1 mutations can also affect ZBP1, which recognizes endogenous Alu element-derived dsRNA, leading to inflammatory transcription [110, 111]. Immune tolerance occurs when cells mark immunogenic dsRNA as “self” through A-to-I RNA editing, thereby preventing an excessive immune response. In esophageal squamous cell carcinoma (ESCC), high-level A-to-I RNA editing mediated by ADAR1 plays a crucial role in blocking dsRNA-triggered immune responses [48, 112]. ADAR2 regulates IL-6 signaling in endothelial cells, recruits circulating immune cells to the vascular endothelium, and participates in immune cell trafficking under ischemic stress lium [37]. As an RNA-binding protein, ADAR3 inhibits A-to-I editing and promotes the expression of the immunoreactive protein MAVS [43].

### RNA editing in cancer

The role of RNA editing in cancer is still not fully understood, although it has long been associated with malignancy [27, 29, 113]. Aberrant expression of ADARs has been observed in various cancers. These include excessive RNA editing due to overexpression of RNA editing enzymes, and reduced RNA editing due to decreased expression of these enzymes [27, 114]. In some cancers, there is no clear correlation between the level of RNA editing and the expression of RNA editing enzymes [25]. The extent of RNA editing and the significance of RNA editing sites vary among different types of cancer. A-to-I RNA editing, which accounts for nearly 90% of all RNA editing events, has been the main focus of studies on the link between RNA editing and cancer, with less research on APOBEC-catalyzed RNA editing in cancer cells [113]. Current understanding suggests that RNA editing plays a crucial role in carcinogenesis through multiple mechanisms.

Changes in the level of RNA editing may lead to alterations in protein sequence and the accumulation of misexpressed proteins within cells, potentially promoting the development of cancer. Therefore, RNA editing may have similar clinical implications to the accumulation of DNA mutations in tumors with a high mutation burden. The DNA damage response serves as a crucial barrier against the malignant transformation of cells, with the DNA repair system playing a key role in maintaining cellular homeostasis. Interestingly, RNA editing has been observed in transcripts of genes involved in DNA repair systems, suggesting that RNA editing may be involved in the initial stages of cancer development. Numerous studies have reported on the impact of ADAR-mediated RNA editing on cell proliferation [115]. For instance, the overexpression of the ADAR gene in non-small cell lung cancer leads to increase A-to-I RNA editing at the K12 site of NEIL1, resulting in a change from an arginine codon to lysine codon at position 242, which is also observed in myeloma [116, 117]. Mutations at this site impair the ability of cells to repair DNA damage caused by oxidative stress. The most common types of RNA editing disorders in cancer cells are enhanced ADAR1 editing and decreased ADAR2 editing, with ADAR1 and ADAR2 being concurrently altered in some cancer cells [118–120]. Increased expression of ADAR1 leads to elevated RNA editing in antizyme inhibitor 1 (AZIN1), resulting in the production fo a recoded AZIN1-S376G protein that impacts cell proliferation [121, 122]. In myeloma, excessive ADAR1 activity leads to the R701G mutation through RNA editing of Glioma-associated oncogene 1 (GLI1) transcription, affecting cancer cell proliferation and resistance to anticancer drugs [123]. While GRIA2 transcripts are normally subjected to RNA editing by ADAR2, decreased editing of GRIA2 transcripts in glioblastoma is associated with downregulation for ADAR2 and altered GluA2 function. Additionally, reduced ADAR2-mediated editing of BLCAP RNA has been observed in bladder cancer, astrocytoma, and colorectal cancer tissues, indicating that ADAR RNA editing is a common and tightly regulated process. In addition to A-to-I RNA editing, C-to-U RNA editing also plays an important role in promoting cancer cell proliferation. Overexpression of APOBEC1 leads to the transition editing of novel APOBEC1 target number 1 (NAT-1) mRNA, resulting in decreased NAT-1 protein expression, which affects the cell cycle [69, 73].

Altered levels of RNA editing in cells can also lead to cytoskeletal damage. For example, in hepatocellular carcinoma and esophageal squamous cell carcinoma, ADAR1 overexpression leads to impaired A-to-I RNA editing at the M2269V site of silk protein B (FLNB), resulting in cytoskeletal changes. Another instance of RNA editing affecting the cytoskeleton is RNA editing at the N136S site of the RhoQ GTPase enzyme RHOQ in colon cancer. RHOQ isoforms generated by RNA editing can impact colon cancer invasion and recurrence by altering the cytoskeleton. RNA editing can also modulate immune responses and immune surveillance in cancer [124]. Furthermore, RNA editing may play a role in cancer by influencing the biogenesis and function of miRNAs that act as tumor suppressors or tumor receptors [125–127]. Recent studies have shown that overexpression of ADAR1 in mice does not initiate or accelerate cancer development, suggesting that ADAR1 overexpression itself is not sufficient to induce cancer but rather a consequence of tumor formation [128, 129]. Therefore, the role of RNA editing in cancer depends not only on the location and level of RNA editing information but also on the specific cancer type [130, 131]. Although many studies have reported the connection between RNA editing and cancer, the mechanism of RNA editing in cancer still remains to be explored. Moreover, RNA editing can expand the repertoire of tumor antigens presented on tumor cells, which can be recognized by the immune system. Furthermore, RNA editing has been described as a process associated with cancer progression, leading to increased cancer growth, invasion, immune evasion, and metastasis. RNA editing could be a target for cancer therapy [110]. Therefore, the study of RNA editing will help us make breakthroughs in cancer immunotherapy and targeted therapy [132].

### The effects of RNA editing on viruses

**Fig. 6:**
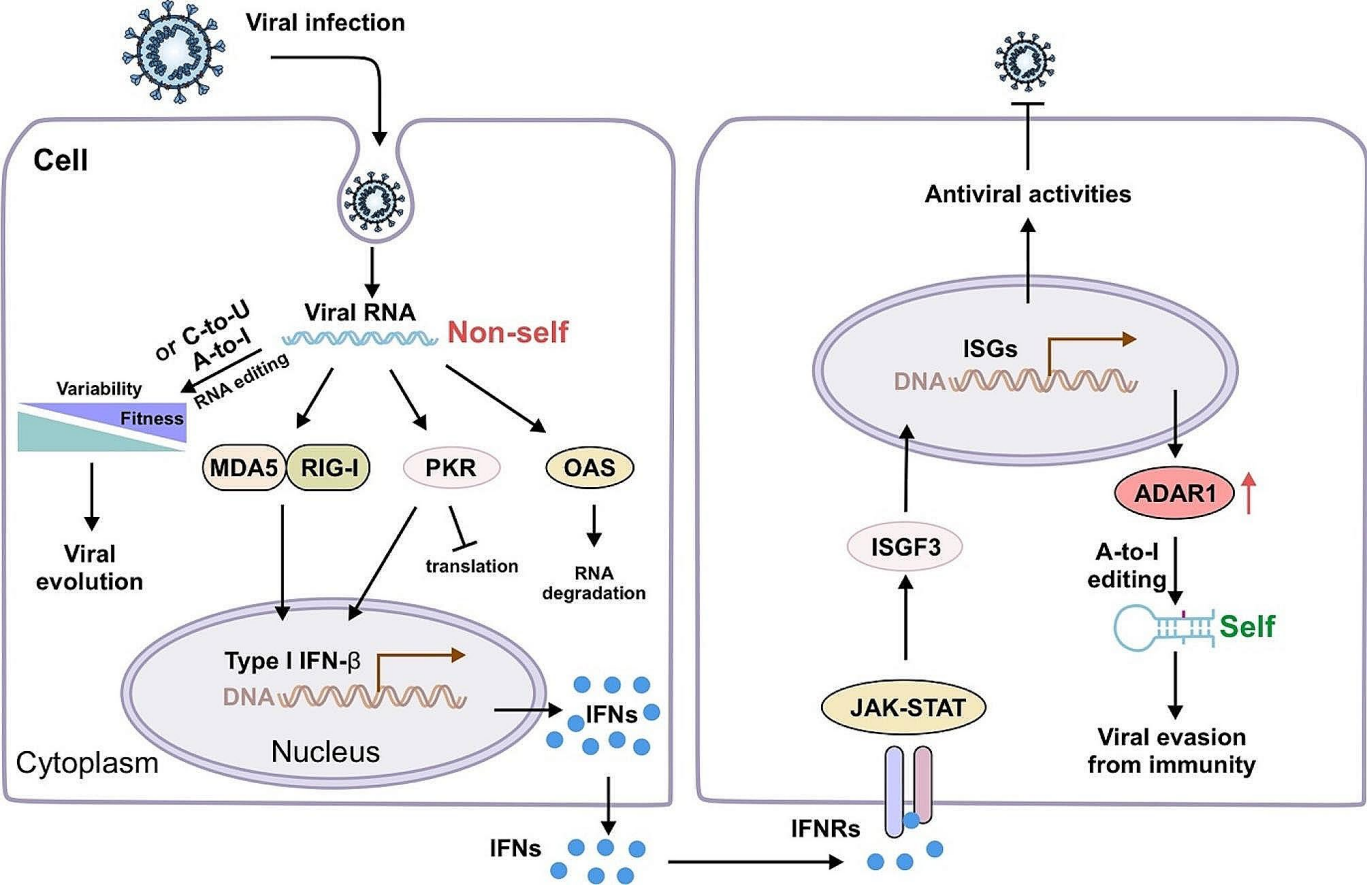
Overview of the possible role of RNA editing in viruses. Viral RNA is recognized by MDA5 and RIG-I and activates downstream signaling pathways. IFN signals are transmitted to other cells through JAK/STAT and ISGF3, inducing ISG transcription, and subsequent antiviral activity. Increased editing of viral RNA by ADAR1 p150 contributes to viral immune evasion. A-to-I or C-to-U RNA editing induce viral genome mutations. A low degree of deamination increases the likelihood of producing viral variants with altered properties

The RNA editing process is involved in regulating of innate immune responses and may play a key role in antiviral defense [44]. RNA editing enzymes can act on viruses through two general mechanisms: RNA editing-dependent and RNA editing-independent [133] (Fig. 6). RNA editing, as a co/posttranscriptional modification, can change nucleotide sequences and have important effects on viral replication, protein synthesis, infection, and virulence. ADARs and APOBECs, in addition to deamination, are RNA-binding proteins that directly interact with endogenous or exogenous RNA to perform biological functions, particularly against viral RNA [69, 134]. Depending on whether deamination occurs during viral infection, the interaction between host RNA editing enzymes and viruses can categorized as cis-regulation or trans-regulation. Trans-regulation implies that ADARs and APOBECs interact with host viral proteins, RNA, or immune factors, without performing deamination functions and participate in host immune response pathways [67]. In both cases, host-mediated RNA editing ultimately affects the viral life cycle, host adaptation, or to some extent the evolutionary direction of the virus [22, 135]. RNA editing in viruses has dual effects. In some cases, it helps the evade the host’s immune system and increases the virus’s fitness. In other cases, it inhibits viral transcription or replication. The dependence on deamination mainly relies on the properties of the virus’s genetic material. Deamination-dependent RNA editing is mostly observed in -ssRNA viruses, while deamination-independent regulation is mainly found in + ssRNA viruses [136]. This difference may be related to the replication mechanism and life cycle of the virus itself. Host RNA editing, in contrast to exogenous RNA, is less efficient in processing exogenous viral RNA. This leads to the stabilization of the dsRNA of the viral RNA, which activates the dsRNA receptor (MDA5) and restores subsequent antiviral effects [33, 96, 101]. Therefore, lack of RNA editing may lead to viral immune evasion as the host has difficulty distinguishing endogenous RNA from viral RNA. However, it is worth noting that viral RNA editing, although on a smaller scale, plays a crucial role in viral evolution. Currently, our understanding of the viral diversity of host RNA editing systems is limited, and further experiments are required to uncover specific mechanisms. The advancement of RNA sequencing technology and the detection of RNA modifications will enable us to gain a more comprehensive understanding of viral RNA editing and discover intriguing phenomena.

## Site-directed RNA editing

Methods to control sequence-specific changes in nucleic acids have become powerful tools in molecular biology and hold promise for the therapeutic correction of disease-causing mutations [4, 137]. Targeting gene mutations or fine-tuning of protein function at the RNA level rather than the DNA level is particularly attractive because RNA editing is reversible and regulatable without permanently altering to the genome [138]. Given that ADARs directly manipulate RNA, there is a growing interest in utilizing ADARs or directing endogenous ADARs to specific adenosines present in mRNA to address disease-associated G to A mutations in the genome [13, 139]. It is estimated that approximately 60% of human disease mutations are caused by SNPs, and RNA editing can correct the most common G-to-A changes, which account for almost 28% of SNPs [140]. In the following section, we will provide a brief introduction to these editing tools and analyze their applications, advantages and disadvantages (Table 1).

**Table 1:** Key examples of site directed RNA editing

Methods	Advantages	Disadvantages	References
A-to-I			
Exogenous ADAR based			
λN-BoxB	The small size allows for adeno-associated virus-based delivery.	Low editing efficiency and some bystander editing.	[38, 141, 142]
SNAP-ADAR	Human origin, small size, chemically stabilized gRNAs are ease to transfect.	It isn’t genetically encodable and unlikely to have therapeutic value.	[143, 144]
WT ADAR2	Simultaneous expression of gRNA and ADAR2 in a single plasmid are enough to manipulate disease-related cellular phenotypes.	Can lead to significant transcriptome-wide off-targeting.	[145]
REPAIR	High editing specificity and easy to viral delivery.	Massive bystander editing.	[146]
Split-ADAR	High editing precision. Tunable and reversible engineering of cellular RNAs for diverse applications.	Interferon response by the delivery modalities.	[147–149]
Bump-Hole	High efficiency and low off-target editing.	With the risk of an antidrug response to the ADAR2 E488Y mutant.	[150]
CIRTS	It is small in size, suitable for efficient viral packaging and delivery. Low propensity to cause immune reactions.	NA	[151, 152]
REWIRE	Small size, entirely originated from human, and can be independently applied to achieve simultaneous A-to-I and C-to-U editing in the same transcript.	The editing efficiency RNAs in animals still needs to be optimized.	[153]
TRIBE	It is beneficial for labelling target RNAs that long-lived interact with RBPs.	The efficiency of single-stranded RNA is reduced, and the substates bias of ADARcd can lead to false negatives.	[154, 155]
Endogenous ADAR based			
RESTORE	The editing is achieved only through the administration of the ASOs.	Some degree of off-target.	[152, 156]
LEAPER	It is safe and the circularization improves the expression level of the gRNA.	A substantial bystander of off-target editing.	[157, 158]
AIMers	Short, chemically modified oligonucleotides can guide efficient and specific RNA editing.	NA	[159]
CLUSTER	High precision RNA editing and the editing homeostasis at natural sites was untouched.	Potential immunogenetic or toxic effects of these highly expressed gRNA species.	[160]
Caged arASO for light triggered RNA editing	Light-triggered RNA point mutation of transcripts in human cells exhibit spatial photoregulation.	Achieving A-to-I editing in CDS of mRNA requires a longer antisense domain.	[161]
RADARS	Specificity, versatility, simplicity, and generalizable across organ systems and species.	The detection of endogenous transcripts showed variable results.	[162, 163]
C-to-U editing			
Exogenous engineered ADAR based			
RESCUE	Expands the RNA targeting arsenal with C-to-U functionality, and easy for delivery	Accidental transcriptome A-to-I deaminaton limit potential therapeutic uses.	[164]
RESCUE-S	Minimize the off-target A-to-I conversions.	Reduced on-target C-to-U editing efficiency.	[165]
SNAP-CDAR-S	Improved the editing of the context of 5’-CCN sequence and improved on-target editing.	There are still notably frequent off-target of A-to-I edits.	[144, 166]
Endogenous APOBEC based			
CURE	Both cytoplasmic and nuclear transcripts could be edited.	The strict codon preference and the potential to induce off-target edits in DNA.	[165]
REWIRE	The editing rate of human cells is high, with a few non-specific editing sites and low levels of off-target globally.	Sequences similar to PUF domain recognition sites may be non-specifically edited.	[153]

NA: not available

### A-to-I RNA editing tools

**Fig. 7:**
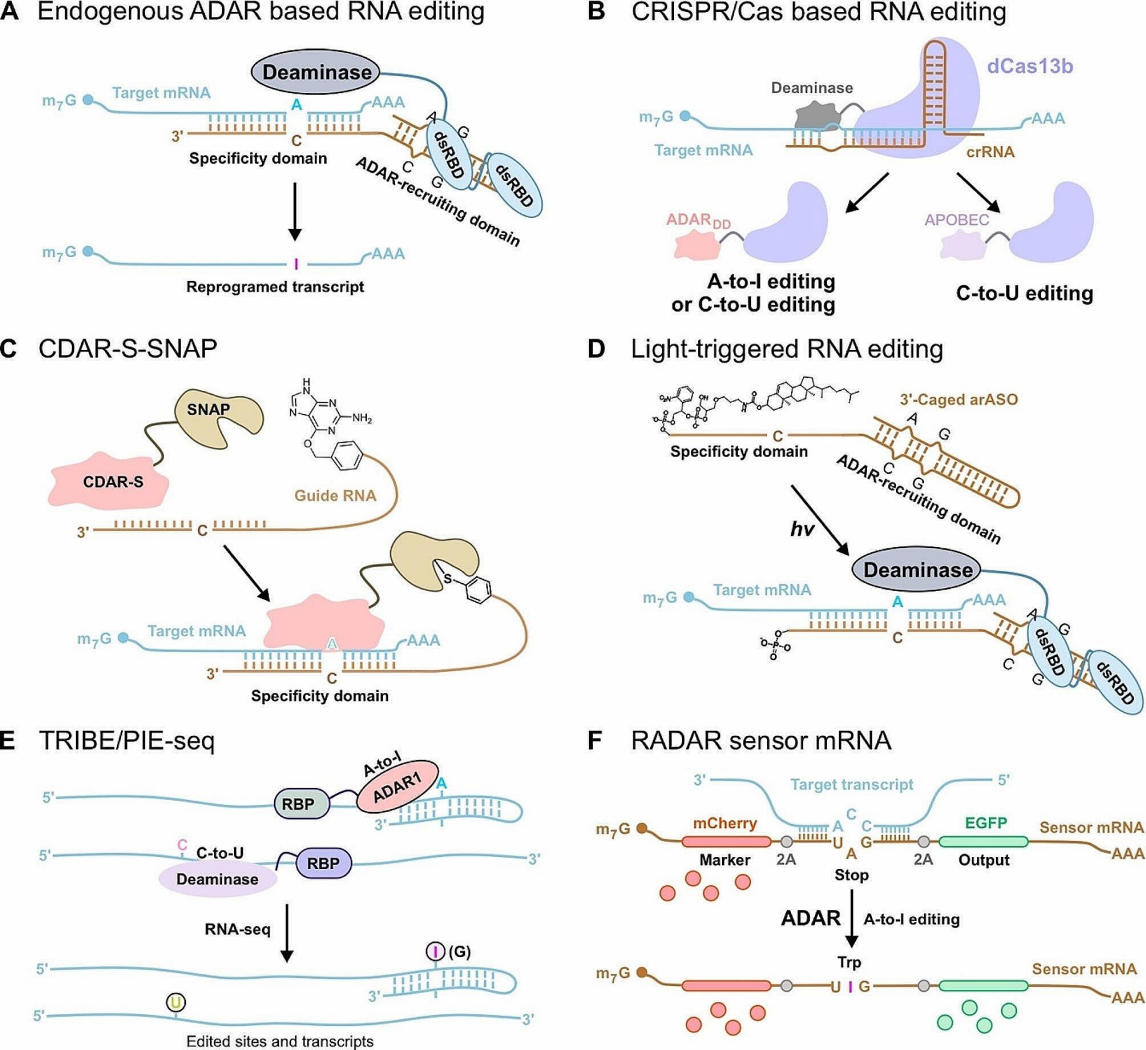
Key examples of the application of RNA editing enzymes. **A** Basic strategy of endogenous ADAR1-mediated site-directed RNA editing. **B** Schematic diagram of the Cas13-based site-directed RNA editing system. **C** The CDAR-S-SNAP tool generates RNA-directed editing enzymes applying self-labeled SNAP-tag. **D** 3’-caged arASO light-triggered RNA editing. Cholesterol modification at the 3’ end prevents photoactivatable antisense guide RNA oligonucleotides from targeting RNA until triggered by light. **E** Schematic representation of the mechanism of TRIBE or PIE sequencing. The deaminase domain introduces a C-to-U or A-to-I editing sites in the sequence adjacent to the RBP binding sites. **F** Schematic of the READR mechanism. The sensor mRNA consists of a 5’ tag domain and a 3’ export domain separated by a 2 A coding region. Base paring between sensor mRNA and target transcript recruits ARARs, which convert the UAG stop codon to a UGG Trp codon, switching on translation of output protein

Although ADARs are usually expressed throughout the body, previous studies have relied on exogenous ADAR enzymes or variants thereof to achieve RNA editing. These systems design the ADAR deaminase domain (ADARdd) to be compatible with chemically modified ADAR-recruiting guide RNAs (adRNAs) or specific secondary structure guide RNAs [167, 168] (Fig. 7). The adRNA portion contains a programmable antisense region that is complementary to the target RNA sequence and plays a role in ADAR recruitment [149, 169]. For programmable RNA editing to work, the RNA-binding element must also be fused to the ADAR protein. For example, fusion of the ADAR deaminase domain to a SNAP-tag allows the deaminase to be covalently attached to a short 5’-O-urapurine-modified guide RNA, causing the guide RNA to localize the deaminase to the target RNA site for A-to-I editing [170]. In addition, ADAR proteins or their deaminase domains are fused to many RNA-binding elements, such as λN-peptide, dCas9 or dCas13 proteins [171–173] (Fig. 7). Subsequently, a smaller molecular weight EcCas6e protein fused to the ADAR deaminase domain was designed [174]. However, off-target effects have always been an important issue in programmed RNA A-to-I editing; ectopic expression of exogenous ADAR fusion proteins increases the risk of off-target RNA editing, while reducing RNA editing makes disease treatment a major challenge. In addition, overexpression of ADARs may lead to additional protein interactions that affect cellular physiology. Therefore, RNA editing tools with high selectivity and operability are needed, and attention has thus been focused on strategies for A-to-I editing using endogenously expressed ADAR proteins.

To address these issues, researchers used engineered adRNAs capable of recruiting endogenous ADARs, so they only needed to provide a single guide RNA that allowed programmable A to I RNA editing [149] (Fig. 7). A disadvantage of this strategy is the relatively low efficiency of RNA editing compared with exogenous delivery of ADARs, which limits its use in biotechnological and therapeutic applications. There is a development strategy called LEAPER (Leveraging endogenous ADAR for programmable editing on RNA) that uses engineered linear adRNAs that can be produced by in vivo expression from viral vectors or chemically synthesized in vitro. Application of LEAPER enables RNA editing and repair of premature stop codons in TP53 of patients with Hurler syndrome. Another strategy is the RESTORE (Recruiting endogenous ADAR to specific transcripts for oligonucleotide-mediated RNA editing) system, which uses chemically modified ADAR recruitment antisense RNA oligonucleotides to achieve A-to-I editing. Based on RESTORE, CLUSTER-adRNA was constructed by adding a cluster of recruitment sequences to achieve more accurate and efficient RNA editing in vitro and in vivo. Existing RNA editing tools are not perfect, so they are being improved in different ways. For example, the short half-lives of gRNAs used in RNA editing tools (especially those without protein binding) limit the efficiency and sustainability of RNA editing. Circular RNA can avoid the degradation of endogenous RNase due to its own stability, so the introduction of engineered ADAR-recruiting guide RNAs (cadRNAs) in RNA editing can improve the efficiency of RNA editing without affecting the accuracy of RNA editing [157, 167]. The method works not only for noncoding regions of cellular transcripts but also for coding regions of RNAs. Further experiments showed that the method is also applicable to in vivo experiments in mice, so that cadRNAs enable highly efficient programmable RNA editing in vivo and have diverse protein regulation and gene therapy applications.

In recent years, different strategies have been used to control the activity of RNA editing enzymes in real time or allow RNA editing enzymes to function in specific regions. The abscisic acid (ABA)-induced RNA editing system enables reversible A-to-I editing in living cells. In addition, the light-triggered A-to-I RNA editing system uses antisense oligonucleotides with a cholesterol modification at the 3’ end. Cholesterol modification had no significant effect on antisense recruitment to the ADAR1 p150 protein, but effectively blocked antisense binding to target RNA fragments, thereby inhibiting A-to-I editing. Light stimulation dissociates the cholesterol moiety from the 3’ end of the antisense molecule and restores the binding of the antisense molecule to the target RNA, enabling A-to-I editing [161]. Although the feasibility of this method has been verified on exogenous mRNA, it is not yet applicable to the CDS region of endogenous transcripts. Further improvements, such as adding longer antisense oligonucleotide regions to edit the CDS region of transcripts, are needed to increase efficiency and fidelity. At the same time, RNA editing enzymes can also be used to develop other biological research tools, such as TRIBE/PIE-seq for detecting RNA-binding protein substrates and RADARS as RNA sensors.

### C-to-U RNA editing tools

Natural RNA cytosine deaminases were discovered long ago, but their high activity toward cytosine present in single-stranded RNA has hindered their application in the development of precision RNA editing. Since the discovery of the Cas13 enzyme, several CRISPR-derived RNA base editing systems have been developed. One example is RESCUE (RNA editing for specific C-to-U exchange) [175], in which a Cas13b variant is fused to ADAR2, a mutation that allows the formation of cytidine deaminase, which converts C-to-U. To address the off-target issue, a high-fidelity variant, RESCUE-S, was developed with additional point mutations but resulted in reduced on-target editing of C-to-U and A-to-I (Fig. 7). More recently, Latifi et al. constructed a new C-to-U RNA editing tool SNAP-CDRA-S, using a cytidine deaminase acting on RNA (CDAR) domain taken from the RESCUE-S tool and a SNAP-tag for RNA targeting [166]. SNAP-CDRA-S reliably provides high on-target products and reduces bystander editing, while the issue of global A-to-I and C-to-U off-target effects remains to be resolved. Although the efficiency and precision of C-to-U RNA editing tools are not yet perfect, these tools represent a critical step in the field of C-to-U RNA editing and open up the possibility of developing tools for other types of RNA editing. APOBEC family proteins with RNA-specific cytidine deaminase activity provide a new tool set for the creation of RNA-specific C-to-U base editors [176] (Fig. 7). RNA editing tools also have room for improvement. It is hoped that in the near future, RNA editing tools with high site specificity, high editing efficiency, easy operation, and no side effects can be applied to the clinical treatment of diseases.

## Conclusion and prospects

As mentioned above, we know much about the role of RNA editing enzymes in biology, but their importance as deaminases and RNA-binding proteins remains to be understood. The precise identification of human ADAR loci remains a challenge, especially at exonic loci of protein-coding mRNAs. Next-generation of RNA sequencing has greatly facilitated the discovery of RNA editing events [17, 177, 178]. In the absence of corresponding sample genome sequencing data, the GIREMI method uses a single short-read RNA-Seq data set to accurately identify RNA editing events [179]. With the development of third-generation sequencing (TGS) technology, long-read RNA sequences are increasingly used to characterize full-length transcripts and can also detect RNA editing sites. L-GIREMI was applied to RNA-seq data from Pacific Biosciences (PacBio) to examine RNA editing sites, allele-specific RNA editing, and region-skipping due to the presence of dsRNA structures in single molecules [180]. Based on Oxford Nanopore Technologies (ONT), the Dinopore method can identify inosine-containing sites in the native transcriptome with high accuracy [181]. The DeepEdit neural network model not only identifies A-to-I editing events in single reads of direct ONT RNA sequencing, but also solves the problem of binning RNA editing events on transcripts [182]. These methods based on multiple RNA-Seq data sets and matching genomic DNA sequencing may generate a large number of false positive signals, and low coverage may be missed after rigorous bioinformatic screening of low coverage RNA-Seq data. Therefore, methods for finding RNA editing sites need further development. Based on the highly selective cleavage activity of endonuclease V on inosine and the universal activity of sodium periodate on all RNAs, the Slic-seq method enriches inosine-containing RNAs and accurately identifies editing sites [18]. REDIportal provides a comprehensive overview of human RNA editing, implements a gene view module to display individual events in genetic context, and hosts the CLARIRE database [15, 177, 183].

However, RNA editing does not tell us all the secrets, and there are many unanswered questions. Despite identifying the positions at which RNA editing occurs, there are still many questions to be answered, such as how RNA editing is regulated, why certain transcripts are edited, and the relationship between RNA editing and diseases [83, 141, 163, 184]. Notably, elucidating the reasons behind tissue and cell-specific A-to-I editing events is imperative, as understanding the variations in editing levels across distinct tissues and developmental stages becomes paramount [16]. Furthermore, advanced research on various aspects of RNA editing enzymes will lead to the development of tools for site-directed RNA editing in RNA therapeutics [24, 29, 90]. Increasing evidence indicates that the extent of RNA editing, the expression of RNA editing enzymes, and specific edited genes are associated with various biological processes and human diseases. Research on the function and regulation of RNA editing enzymes can not only improve our understanding of diseases, but also provide valuable insights into the precise treatment of RNA editing-related diseases. RNA editing tools, which are based on the enzymatic activity of RNA editing enzymes, offer distinct advantages. Unlike genome editing technologies (e.g., DNA editing), RNA editing technology modifies only the RNA without altering the genome sequence, thereby minimizing safety and ethical concerns [142]. In addition, RNA editing technology is technically simpler and more acceptable to patients than DNA editing. Therefore, this technology has great potential and feasibility in clinical treatment of genetic diseases. However, current RNA editing tools still have certain limitations that need to be addressed before their clinical applications. Firstly, comprehensive research on RNA editing enzymes is essential to improve editing efficiency, minimize immune response, and reduce side effects. Secondly, targeted delivery strategies of RNA editing enzymes need to be optimized to improve editing efficiency and minimize off-target effects. Utilizing endogenous RNA editing enzymes for RNA editing can optimize RNA editing platforms by reducing the reliance on exogenous and immunogenic protein-directed editing. Additionally, exploring the regulation of protease active centers or protein-protein interaction interfaces can provide better control over the function of RNA editing enzymes. Overall, advancements in the field provides confidence that safe and effective RNA editing tools can be developed and utilized for the treatment of a wide range of diseases.

## Data Availability

Not applicable.

## Abbreviations

A-to-I: Adenosine to inosine
ADAR: Adenosine deaminases acting on RNA
ADATs: Adenosine deaminases acting on transfer RNAs
adRNAs: ADAR-recruiting guide RNAs
AGS: Aicardi-Goutières syndrome
AICDA: Activation induced cytosine deaminase
ApoB: Apolipoprotein B
APOBEC: Apolipoprotein B mRNA editing catalytic polypeptide-like family
C-to-U: Cytosine to uridine
CDAR: Cytidine deaminase acting on RNA
CDS: Coding sequence
CIRTS: CRISPR-Cas-Inspired RNA Targeting System
CURE: C > U RNA Editor
dsRBDs: DsRNA-binding domains
eIF2α: Eukaryotic initiation factor 2α
ESCC: Esophageal squamous cell carcinoma
GluA2: Glutamate ionotropic receptor AMPA-type subunit 2
IFN: Interferon
LEAPER: Leveraging endogenous ADAR for programmable editing on RNA
MAVS: Mitochondrial antiviral-signaling protein
MDA5: Melanoma differentiation-associated gene 5
NAT-1: Novel APOBEC1 Target number 1
NES: Nuclear export signal
NLS: Nuclear localization signal
OAS: Oligoadenylate synthetase
PKR: Protein kinase R
RADARS: Reprogrammable ADAR sensors
REPAIR: RNA Editing for Programmable A to I Replacement
RESCUE: RNA Editing for Specific C to U Exchange
RESTORE: Recruiting endogenous ADAR to specific transcripts for oligonucleotide-mediated RNA editing
REWIRE: RNA editing with individual RNA-binding enzyme
RIG-I: Retinoic acid-inducible gene I
SCD1: Stearoyl-CoA desaturase 1
TadA: Bacterial tRNA deaminase
TLR: Toll-like receptors
TRIBE: Targets of RNA-binding proteins identified by editing
ZBP1: Z-DNA binding protein 1
